# The Research Defence Society

**Published:** 1908-05-02

**Authors:** 


### The Hospital

A Journal of

The Medical Sciences and Hospital Administration.

New Series. No. 59, Vol. III. [No. 1131, Vol. XLIV.] SATURDAY, MAY 2, 1908.

### The Defence of Research.

During the early part of March we published a short notice of the formation of a Research Defence Society for the purpose of making known the facts with regard to experiments on animals in this country. In the present number we print a letter from Lord Cromer, which explains the objects of this Society and indicates in general terms the methods which it proposes to adopt. The President's letter sets forth very shortly some of the more striking benefits which have resulted to mankind from experiments upon animals. With these and many other advances in the comprehension and treatment of disease which are the outcome of what is termed "vivisection" the medical profession is familiar. But the firm and almost unanimous opinion of the medical profession upon this subject, supported by an occasional weighty pronouncement in the more respectable of the daily papers, is not enough. Year by year the benefits following experimental work have been more and more widely diffused, until those who daily make use of the results of scientific research for the good of their patients have almost come to take them for granted, and to suppose that time and the spread of education will bring about the enlightenment of the public. Meanwhile the opponents of the experimental method have pursued unceasingly their work of detraction. Many of them are doubtless inspired by motives which we cannot help respecting, however much we may deplore that strange narrowness of vision which perverts their judgment and reconciles them to the lowest tricks of controversy. But whatever may be the motives of antivivisectionists, and whatever our opinion of their methods may be, there can be no doubt that their continued efforts have already succeeded in poisoning the minds of a considerable section of the public. Extravagant statements which at one time were treated with contemptuous indifference are now listened to and perhaps accepted. Should this campaign of misrepresentation proceed in the near future with as little organised opposition as there has been in the past, the result to medical progress, and therefore to the welfare of mankind, must be disastrous.

The formation of the Research Defence Society is therefore most opportune. The antivivisection movement has indeed been allowed to go on too long without active systematic opposition. The new Society, now that it has come into existence, must be supported all the more vigorously because the need for it is urgent. In Lord Cromer the Society has a president whose administrative ability is undoubted; the list of vice-presidents includes the names of men distinguished in every department of public life; while Mr. Stephen Paget's name is a guarantee that the Society will not lack skill and enthusiasm in the organisation of its campaign. The membership is already more than 800.

We would urge our readers to study Lord Cromer's letter with care, and, whether they decide to join the Society or not, to take some part in the furtherance of its objects. Every medical man is the centre of a circle of public opinion, however small, and has it in his power to neutralise to some extent the insidious poison which has been instilled into the public mind. Many patients, weary of the conflicting appeals which are made to their heads and their hearts, and bewildered by contradictory statements, are impelled to ask their medical attendants for help in their difficulty. It is our duty to be prepared with a reasoned answer to such inquiries. The dogmatic method, too long adopted, must be cast aside. Experiments upon animals do not naturally justify themselves to the lay mind. It does not satisfy our patients to learn that we ourselves are convinced of the absolute necessity for such experiments and of the immense benefit that has already resulted from them; we must be ready and willing to define our position and to advance our reasons. That is a compliment which the public has now learnt to expect from us, and we shall injure the cause of research and the reputation of the profession if we refuse to pay it.

But the programme of the Research Defence Society ensures more than the instruction of the educated classes in the true facts of experimental research, and the establishment of an information bureau for all those interested in the subject. The Society proposes to bring home to the popular understanding "the case for vivisection" by means of lectures, leaflets, and speakers skilled in the art of debate. The *Morning Post*, which like the *Times* devotes an able and well-informed leading article to this topic, suggests that the harrowing pictures placed in railway stations should be answered by pictures of human beings and animals suffering from some of the awful diseases from which treatment founded upon experimental methods has now saved them. This may not be practicable, but it recognises the principle that the Society must sometimes fight its opponents by using their weapons. The antivivisectionists must be attacked at every point at which they have established a position. Although they are already divided into factions bitterly opposed to each other, their strength and influence are considerable, and the Research Defence Society has a hard task before it. It will be our duty and our pleasure to assist the Society to the best of our powers throughout its operations. The matter does not merely concern the progress of science, but affects the future welfare of humanity, and each of us must do our share of the work.

